# Early identification and awareness of child abuse and neglect among physicians and teachers

**DOI:** 10.1186/s12887-024-04782-3

**Published:** 2024-05-04

**Authors:** M. Roeders, J. Pauschek, R. Lehbrink, L. Schlicht, S. Jeschke, M.P. Neininger, A. Bertsche

**Affiliations:** 1 University Hospital for Children and Adolescents, Neuropaediatrics, 17475 Ferdinand-Sauerbruch-Str. 1, Greifswald, Germany; 2 University Hospital for Children and Adolescents, Neuropediatrics, 18057 Ernst-Heydemann-Straße 8, Rostock, Germany; 3 Pediatric Clinic Bonifatius Hospital Lingen, 49808 Wilhelmstraße 13, Lingen, Germany; 4 Clinical Pharmacy, Institute of Pharmacy, Medical Faculty, Leipzig University, and Drug Safety Center, Leipzig University and Leipzig University Hospital, 04103 Bruederstrasse 32, Leipzig, Germany

**Keywords:** Child abuse, Child neglect, Awareness, Physicians, Teachers, Early identification

## Abstract

**Background:**

Child abuse and neglect (CAN) causes enormous suffering for those affected.

**Objective:**

The study investigated the current state of knowledge concerning the recognition of CAN and protocols for suspected cases amongst physicians and teachers.

**Methods:**

In a pilot study conducted in Mecklenburg-Western Pomerania from May 2020 to June 2021, we invited teachers and physicians working with children to complete an online questionnaire containing mainly multiple-choice-questions.

**Results:**

In total, 45 physicians and 57 teachers responded. Altogether, 84% of physicians and 44% of teachers were aware of cases in which CAN had occurred in the context of their professional activity. Further, 31% of physicians and 23% of teachers stated that specific instructions on CAN did not exist in their professional institution or that they were not aware of them. All physicians and 98% of teachers were in favor of mandatory training on CAN for pediatric residents and trainee teachers. Although 13% of physicians and 49% of teachers considered a discussion of a suspected case of CAN to constitute a breach of confidentiality, 87% of physicians and 60% of teachers stated that they would discuss a suspected case with colleagues.

**Conclusion:**

Despite the fact that a large proportion of respondents had already been confronted with suspected cases of CAN, further guidelines for reporting procedures and training seem necessary. There is still uncertainty in both professions on dealing with cases of suspected CAN.

**Supplementary Information:**

The online version contains supplementary material available at 10.1186/s12887-024-04782-3.

## Introduction

Child abuse and neglect (CAN) is a global problem [1]. It is estimated that incidents of different types of sexual abuse vary from 8 to 31% for girls and from 3 to 17% for boys throughout the world [2]. In Germany, more than 59,900 children and adolescents were identified as being at risk of neglect, and psychological, physical, or sexual violence in the year 2021 [3]. Child abuse and neglect are a widespread problem. The number of unreported cases is estimated to be high. The officially reported cases of CAN are below the 1% limit. However, retrospective surveys of young people and adults indicate a lifetime prevalence of more than 10% [4].

CAN is a problem on several levels. It is well known that the long-term consequences of CAN are severe and often persistent [5]. For example, affected children have a higher risk for developing an internalizing and externalizing mental disorder, drug abuse, suicide attempts, sexually transmitted infections, and risky sexual behavior, or, once they are adults, to abuse their own children [6–9].

In addition to the enormous burden for the individual, there are economic consequences for society. The annual costs of CAN are estimated to range from 11.1 to 29.8 billion Euros in Germany per year [10] and up to 124 billion US Dollars in the US [11]. Studies suggest that a 10 per cent decrease of CAN prevalence in America and Europe could lead to annual savings of 105 billion dollars [12]. Thus, CAN carries burdens both for the affected individual and society.

Despite the high prevalence and resulting consequences, studies regarding early identification and action procedures are rare [13]. In addition, some issues have not been adequately addressed in professional groups such as teachers and physicians. Members of these professions are usually the adults outside the family who are in closest and most frequent contact with children. They therefore have the potential to play an essential part in the identification of CAN [14] and should be adequately trained [15]. Nevertheless, even for those professionals the detection of CAN often remains difficult [16]. As both physicians and teachers play an important role in child protection, it is essential to investigate their current level of understanding of CAN and their strategies for dealing with suspected cases. In this way, an accurate baseline for training can be established, in order that these professions be adequately qualified to intervene early in cases of CAN. We therefore performed a pilot study in Mecklenburg-Western Pomerania surveying teachers and physicians about their current knowledge of CAN, actions taken in case of a CAN, existing protocols in their institutions, training on the topic in the past and their individual need for further training. We aimed to compare the knowledge and information needs of those two professional groups, which both can act as key players in the detection of CAN. In the long term, those insights will enable the development of new training programs, or the improvement of those that already exist.

## Materials and methods

### Setting and participants

After approval of the ethics committee of the Medical Faculty of Rostock University and the Rostock school authority, we conducted this pilot study with physicians and teachers from May 2020 to June 2021. In total, three hospitals as well as eight schools in Mecklenburg-Western Pomerania agreed to participate in the pilot survey. Two university hospitals and one primary care hospital were included in the study. In total, these hospitals reflect around 30% of the bed capacity for pediatric and adolescent medicine in Mecklenburg-Western Pomerania. It can be assumed that the number of physicians in the departments contacted was around 70, resulting in a response rate of around 65%. Clinic directors and principals of schools received an e-mail containing an information sheet and a link to access the questionnaire and were asked to spread the e-mail in their teams.

The survey was accessible without registration or a password. The link provided in the invitation e-mails led directly to the questionnaire. For data collection we used EvaSys, a software for conducting surveys [evasys V9.0 (2404), EvaSy GmbH]. Participants were informed about the study objectives, voluntary participation, and anonymization in the questionnaire introduction. The participants were informed that by filling in the questionnaire they agreed to participate in the study.

### Questionnaire

A study team consisting of pediatricians and medical students interested in the topic of CAN designed the questionnaire. Current literature was researched during the development of the questionnaire. To improve comprehensibility, clarity, and readability, the questionnaire was pre-tested with 10 physicians and 10 teachers. After the pre-test, the physicians and teachers were interviewed about the questionnaire. This was followed by further adjustments to optimize the questionnaire.

The questionnaire consisted of single-choice, multiple-choice, and Likert-scale answering options. After a short introduction, the participants were asked questions on the following topics (Supplement 1):

1. Personal experiences of physicians and teachers regarding the topic of CAN.
2. Knowledge about the topic of CAN, including bruising patterns.
3. Actions taken in case of a CAN and existing protocols in their institutions in cases of suspected CAN.
4. Confidentiality and associated difficulties in reporting procedures.
5. Training on the topic of CAN.

Additionally, sociodemographic data were collected at the end of the questionnaire.

With regard to the question of bruising patterns, the body parts were adapted to the TEN-4-FACESp Bruising Rule [17].

### Statistics

Calculations were performed using SPSS (Statistical Package for the Social Sciences, Version 26, IBM Corporation, Armonk, New York, USA). Frequencies are reported as numbers and percentages. For further statistical analysis, we applied Chi-square tests, Fisher’s exact tests and Mann-Whitney-U-tests as appropriate. A *p*-value ≤ 0.05 was considered to indicate significance.

## Results

### Participants

In total, 45 physicians and 57 teachers took part in the survey. Sociodemographic data summarized in Table 1.

**Table 1:** Sociodemographic data of the interviewed physicians and teachers. Total respondents: Physicians *n* = 45, Teachers *n* = 57

Participants	Physicians *n* (%)	Teachers *n* (%)
Gender		
Male	14 (31%)	10 (18%)
Female	30 (67%)	45(79%)
No answer	1 (2%)	2 (3%)
Age		
Under 30 years old	10 (22%)	6 (11%)
Between 30 and 50 years old	28 (62%)	29 (51%)
Over 50 years old	7 (16%)	22 (39%)

Of teachers, 13/57 (23%) reported working at an elementary school, 10/57 (18%) at a school for children with special needs, 24/57 (42%) at a grammar school, and 10/57 (18%) at an other secondary school.

### Personal experiences of physicians and teachers regarding child abuse and neglect

Of the participants, 38/45 (84%) physicians and 25/57 (44%) teachers reported that they had been confronted with cases of CAN in the past. None of the physicians, and 10/57 (18%) of teachers stated that they were unsure about whether they had witnessed a case of CAN before. Of physicians, 7/45 (14%) and of teachers, 22/57 (38%) reported that they had not been involved in any cases of CAN so far.

Physicians (32/45; 71%) reported more frequently having already encountered a case of physical abuse compared to teachers (15/57; 26%, *p* < 0.001). Also, experiences with cases of physical neglect were reported more frequently by physicians (32/45; 71%) than by teachers (13/57; 23%; *p* < 0.001). More details are shown in Table 2.

**Table 2:** Reported experience with cases of various forms of child abuse and neglect (CAN). Multiple answers were possible. Total respondents: Physicians *n* = 45, Teachers *n* = 57

Form of CAN	Physicians *n* (%)	Teachers *n* (%)	*p*-value
Physical abuse	32 (71%)	15 (26%)	< 0.001
Physical neglect	32 (71%)	13 (23%)	< 0.001
Emotional neglect	27 (60%)	26 (46%)	n.s.
Emotional abuse	19 (42%)	19 (33%)	n.s.
Sexual abuse	16 (36%)	8 (14%)	0.010

### Knowledge about child abuse and neglect

#### Knowledge of typical signs and behavior patterns

**Fig. 1:**
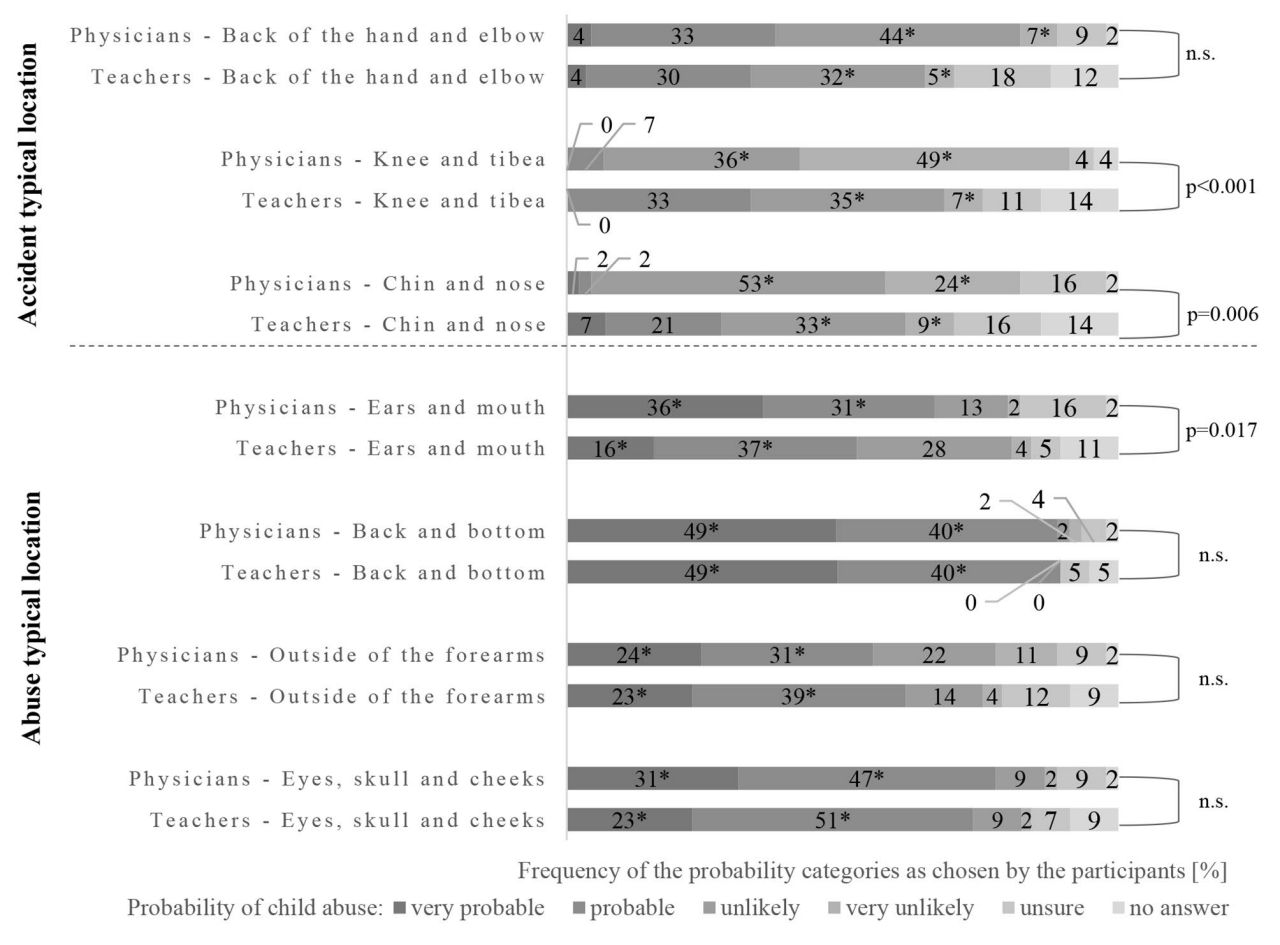
Legend to Fig. 1: Respondents’ answers on the probability of child abuse if the respective body parts were affected. Respondents could indicate probabilities on a Likert scale ranging from very probable to very unlikely Correct answers are marked with *. Total respondents: Physicians *n* = 45, Teachers *n* = 57

Of the participants, 40/45 (89%) physicians and 51/57 teachers (89%; n.s.) correctly assumed that the back and bottom are very likely body sites for signs of CAN. In addition, 35/45 (78%) physicians and 24/57 (42%; *p* < 0.001) teachers correctly indicated that injuries to the chin and nose were unlikely indicators of possible CAN. Further information is presented in Fig. 1.

Asked for their opinions on what emotional abnormalities could occur in the context of CAN, 41/45 (91%) physicians and 43/57 (75%; *p* = 0.039) teachers indicated that verbal and socioemotional developmental delays might occur. More details are shown in Table 3.

**Table 3:** Emotional abnormalities most likely to be associated with child abuse and neglect (CAN). Multiple answers were possible. Total respondents: Physicians *n* = 45, Teachers *n* = 57

Emotional abnormalities most likely to be associated with CAN	Physicians *n* (%)	Teachers *n* (%)	*p*-value
Aloof or withdrawn behavior	43 (96%)	52 (91%)	n.s.
Verbal and socioemotional developmental delays	41 (91%)	43 (75%)	0.039
Early childhood depression	29 (64%)	33 (58%)	n.s.
Lack of interest and involvement	28 (62%)	39 (68%)	n.s.
Conformist behavior	24 (53%)	25 (44%)	n.s.

When asked which parental behavior could most likely indicate CAN, 41/45 (91%) physicians and 44/57 (77%; n.s.) teachers responded slight irritability and overwhelming demands. Further, 40/45 (89%) physicians and 31/57 (54%; *p* < 0.001) teachers reported inappropriate reactions (exaggerated or underexaggerated) as indicators for CAN (Table 4).

**Table 4:** Parental behaviors that are most likely to indicate possible child abuse or neglect (CAN) according to the participating physicians and teachers. Multiple answers were possible. Total respondents: Physicians *n* = 45, Teachers *n* = 57

Parental behaviors most likely to indicate possible CAN	Physicians *n* (%)	Teachers *n* (%)	*p*-value
Irritability and overwhelming demands	41(91%)	44 (77%)	n.s.
Inappropriate reactions (exaggerated or underexaggerated)	40 (89%)	31 (54%)	< 0.001
Uncooperative parental behavior	32 (71%)	35 (61%)	n.s.
Over-adapted behavior	22 (49%)	23 (40%)	n.s.
Other	7 (16%)	1 (2%)	0.010

#### Estimated long-term effects

Of physicians, 37/45 (82%) and 42/57 (74%; n.s.) teachers assumed that children who have had experiences of abuse and/or neglect might behave similarly towards their own children in the future.

42/45 (93%) physicians and 52/57 (91%; n.s.) teachers anticipated long-term consequences for affected children due to a failure to report CAN.

### Actions and instruction procedures in cases of suspected child abuse and neglect

Where CAN was suspected, physicians (39/45; 87%) would discuss the case with colleagues more frequently than teachers (34/57, 60%; *p* = 0.003). More details on actions physicians and teachers would consider are shown in Table 5.

**Table 5:** Answers of physicians and teachers to the question how they responded or would have responded in a possible case of child abuse and neglect (CAN). Multiple answers were possible. Total respondents: Physicians *n* = 45, Teachers *n* = 57

Responses related to cases of CAN	Physicians *n* (%)	Teachers *n* (%)	*p*-value
Discuss with colleagues	39 (87%)	34 (60%)	0.003
Request a forensic medical consultation (response option only given to physicians)	35 (78%)	-	-
Inform the authorities	27 (60%)	22 (39%)	0.03
Interview the child	22 (49%)	25 (44%)	n.s.
Confront parents/relatives	13 (29%)	20 (35%)	n.s.

To the question whether specific instructions regarding responses to suspected CAN were in place, 31/45 (67%) physicians and 44/57 (77%) teachers answered, “yes, there are specific instructions in my institution”; 5/45 (11%) physicians and 1/57 (2%) teachers answered, “no, there are no specific instructions in my institution”; and 9/45 (20%) physicians and 12/57 (21%) teachers answered, “I am not aware of specific instructions in my institution”. 42/45 (93%) of physicians and 48/57 (84%; n.s.) of teachers said that a generally applicable guideline for dealing with suspected cases of CAN could have a positive effect.

### Impact of confidentially

When asked whether the duty of confidentiality influenced the respondents in their actions, 14/45 (31%) physicians and 23/57 (40%; n.s.) teachers agreed.

When asked in which scenarios a breach of confidentiality occurs according to the participants, 6/45 (13%) physicians and 28/57 (49%; *p* < 0.001) teachers assumed that sharing information with colleagues constituted a breach. 17/45 (38%) of physicians, and 7/57 (12%; *p* = 0.003) of teachers, assumed that passing on information to the police would be a breach of confidentiality. Multiple answers were possible. Further results are displayed in Table 6.

**Table 6:** Respondents’ answers to the question of when a breach of confidentiality has occurred. Multiple answers were possible. Total respondents: Physicians *n* = 45, Teachers *n* = 57

Disclosures to the following professionals constitutes a breach of confidentiality	Physicians *n* (%)	Teachers *n* (%)	*p*-value
Police	17 (38%)	7 (12%)	0.002
Youth Welfare Office	16 (36%)	13 (23%)	n.s.
Colleagues	6 (13%)	28 (49%)	< 0.001
Parents	5 (11%)	22 (39%)	0.001
Other	16 (36%)	15 (26%)	n.s.
None of the above	14 (31%)	7 (12%)	0.019

### Training on the topic of child abuse and neglect

2/45 (4%) of physicians reported that they felt that CAN was a taboo subject in the professional setting, compared to 10/57 (18%; *p* = 0.041) of teachers. However, both professional groups (physicians: 34/45, 76%; teachers: 46/57, 81%; n.s.) supported the importance of increasing public awareness of the issue.

In terms of training, 43/45 (96%) of physicians and 40/57 (70%; *p* = 0.001) of teachers reported that they had already attended training on CAN during or after their studies (multiple answers were possible). 43/45 (96%) of physicians, and 53/57 (93%; n.s.) of teachers, confirmed the importance of continuous education to deepen knowledge, including after the completion of their studies.

The idea that the topic of CAN should be a mandatory part of the training for pediatricians and teachers was supported by 45/45 (100%) of physicians and 56/57 (98%; n.s.) of teachers.

Among physicians, 32/45 (71%) reported feeling adequately informed about the topic compared to 21/57 (37%; *p* < 0.001) of teachers. 34/45 (76%) of physicians, and 22/57 (39%; *p* < 0.001) of teachers, still required more information regarding CAN.

When asked by whom the information should be provided, the following professional groups were mentioned: psychologists (physicians: 11/45, 24%; teachers: 30/57, 53%; *p* = 0.004), the youth welfare office (physicians: 1/45, 2%; teachers: 12/57, 21%; *p* = 0.005), physicians (physicians: 22/45, 49%; teachers: 7/57, 12%; *p* < 0.001), authorities such as the health department (physicians: 1/45, 2%; teachers: 0/57, 0%; n.s.), schools (physicians: 1/45, 2%; teachers: 2/57, 3%; n.s.), and others (physicians: 5/45, 11%; teachers: 3/57, 5%; n.s.).

## Discussion

This study gives insights into the awareness and handling of CAN among physicians and teachers. An overall majority of respondents reported previous experiences with cases of CAN. Remarkably, both professional groups reported uncertainty in dealing with cases of CAN and did not feel sufficiently prepared to report such cases.

In this study, 84% of the physicians and 44% of the teachers reported previous experiences with CAN.

It is noteworthy that none of the physicians interviewed was unsure whether he or she had ever been confronted with a case of CAN. In contrast, some teachers were unsure. Teachers experience the children and families in many sometimes contradictory facets over long periods of time and thus have more opportunities to reflect on and question their own judgements.

The higher level of exposure of physicians compared to teachers may explain why, in this questionnaire, they tended to show greater theoretical and practical knowledge than teachers regarding the forms and signs of CAN. A large proportion of physicians was exposed to suspected cases of physical abuse and neglect. Those teachers who reported having been confronted with CAN were more likely to have exposure to suspected cases involving emotional abuse and neglect. Those results might be explained by the different professional activities of physicians and teachers. Both physical and emotional types of CAN can have long-term consequences such as mental disorders, drug abuse, suicide attempts, sexually transmitted infections, and risky sexual behavior [8]. Further, children whose mothers were abused as children are at high risk of being abused themselves, thus creating a vicious cycle [9]. For this reason, it is important to identify CAN as early as possible in order to be able to intervene and reduce the risk of long-term consequences. The majority of both professions were aware of long-term consequences as well as possible early warning signs, especially in specific patterns of parental behavior. This result can be seen as very positive since in both groups theoretical knowledge is present to identify not only CAN but also to detect early warning signs of CAN. Our study shows that physicians generally feel better informed than teachers. It is therefore surprising that a higher percentage of physicians than teachers would still like to have more information on the topic of CAN. It can be assumed that their wider direct experience with cases of CAN makes them aware of gaps in knowledge and thus leads to the demand for a guideline with concrete instructions for action and further training.

Nevertheless, both professions agreed that the topic of CAN should be a mandatory part of training which should also be regularly refreshed. Mandatory courses designed to train in the early identification and intervention of CAN significantly increase knowledge and self-awareness of this topic [15] and thus increase the detection and encourage the reporting of cases of CAN.

Instructions and legal frameworks can make it easier for physicians and teachers to identify CAN and to deal with reporting procedures. Thus, those measures are amongst the most important interventions to prevent CAN [18]. When asked if their facilities had specific instructions for suspected CAN, our study shows that many physicians and teachers were not adequately informed. A survey showed that the majority of healthcare professionals in Germany did not feel confident in applying the Child Welfare Law [19], and felt insecure about the legal framework and its application [19]. Similar results were also seen in this study, although most physicians stated to have received training on this topic during their university studies or pediatric training. Consequently, the share of physicians who received training is much higher than in other studies. However, as many physicians still report uncertainties the current standard training is insufficient. Respondents were uncertain in which cases a breach of confidentiality occurs. This creates a barrier that can lead to delayed reporting or even non-reporting behavior [20]. Significantly more teachers than physicians assumed that disclosure to colleagues is associated with a breach of confidentiality. Most physicians would discuss a possible suspicion with colleagues; significantly fewer teachers would do so. This contradicts a guideline issued in Germany in 2019 that provides recommendations for diagnostics and management in child protective services [21]. This guideline states that physicians and other professionals are strongly advised to seek the help of experienced professionals when CAN is suspected. This indicates that the guideline is not yet sufficiently known and not routinely applied. In addition, it is important to make counselling and support services for professionals even better known. Professionals and private individuals have the opportunity to obtain low-threshold help from the Mecklenburg-Western Pomerania child protection hotline, child and youth welfare professionals, forensic medicine institutes and the federal medical child protection hotline, for example.

Overall, it can be concluded that an obligation to participate in further training is necessary, or that these trainings must be more attractive. Mandatory and recurring courses for example using case studies and simplified checklists should therefore be discussed. Tools such as screening instruments also need to be further investigated and established. Recent studies show that such implementations, combined with adequate training and concise action procedures, can increase the early identification of CAN [22].

Since child protection is a multi-professional task, the training should also be carried out by different professional groups as they have different resources and contact points with affected families at different times [23]. In many cases of CAN it is important to work together systematically and interdisciplinary, and to use different expertise, skills and resources. It is therefore important that interdisciplinary work is practiced in training and that opportunities for networking between different professional groups are encouraged.

### Limitations

As participation was voluntary, it can be assumed that motivated professionals with a connection to the topic of child protection, both among physicians and teachers, are more likely to have responded. Thus, uncertainty in dealing with CAN may be greater than reported.

Due to the high workload caused by the Covid-19 pandemic, only eight schools and three hospitals agreed to participate. In order to address the important issue of CAN nonetheless, we decided to start a pilot survey in these schools and hospitals. As another consequence of the Covid-19 pandemic, the surveys could not be conducted in person as originally planned. Due to the lack of personal visits, a lower response rate is assumed.Although different results of the professional groups in the survey on physical abnormalities were to be expected, the same questions were asked in each case for better comparability. This made it possible to confirm the different expertise and emphasize the indispensability of both professions for the identification of CAN.

## Conclusion

In summary, although most physicians and teachers report professional experience with cases of CAN, many of them display uncertainty in dealing with suspected cases. Clear, concise institutional guidelines for dealing with CAN are needed as well as support for teachers and physicians confronted with suspected cases of CAN.

### Electronic supplementary material

Below is the link to the electronic supplementary material.

Supplementary Material 1

## Data Availability

The datasets used and/or analysed during the current study available from the corresponding author on reasonable request.

## Abbreviations

CAN: Child abuse and neglect
