# Supplementary material for: Early identification and awareness of child abuse and neglect among physicians and teachers
Source: BMC Pediatr. 2024 May 4;24:302. doi: 10.1186/s12887-024-04782-3 (PMC11069270; doi:10.1186/s12887-024-04782-3)
Supplement: Supplementary file 1 — Supplementary Material 1 [file 12887_2024_4782_MOESM1_ESM.docx]

# Questionnaire

| Early identification and awareness of child abuse and neglect among physicians and teachers  The original questionnaire was conducted in German. For a better understanding of the survey, we translated the original questionnaire. | |
| --- | --- |
| Question | Answer |
| Gender | 1. female 2. male 3. other |
| Age | 1. < 30 years old 2. 30 – 50 years old 3. > 50 years old |
| For teachers: „In which kind of school do you work? | 1. elementary school 2. school for children with special needs 3. grammar school 4. other secondary school |
| Have you personally been confronted with any cases in which child abuse or neglect have occurred? | 1. yes 2. no 3. unsure |
| If yes, what form of child abuse and neglect occurred in this case? Multiple answers possible | 1. physical abuse 2. physical neglect 3. emotional abuse 4. emotional neglect 5. sexual abuse |
| On which parts of the body it is likely that the injuries were caused by violence?  *Options per item:*   - *very probable,* - *probable,* - *unlikely,* - *very unlikely,* - *unsure* | 1. back of the hand and elbow 2. knee and tibea 3. chin and nose 4. ears and mouth 5. back und bottom 6. outside of the forearms 7. eyes, skull and cheeks |
| What are the most likely emotional abnormalities that might stand out in the context of child abuse and neglect? Multiple answers possible | 1. delay in verbal and socioemotional development 2. lack of interest and involvement 3. aloof or withdrawn behavior 4. early childhood depression 5. conformist behavior 6. other |
| What parental behavior most likely indicates child abuse and neglect? Multiple answers possible | 1. uncooperative parental behavior 2. inappropriate reactions 3. irritability and overwhelming demands 4. over-adapted behavior 5. other |
| Do you think that children who have experienced child abuse and/ or neglect might behave similarly towards their own children in the future? | 1. yes 2. no 3. unsure |
| Do you think that failure to report child abuse and neglect can cause long-term consequences for the affected children? | 1. yes 2. no 3. unsure |
| How would you respond to a suspicion of child abuse and neglect? Multiple answers possible | 1. discuss with colleagues 2. confront parents or relatives directly 3. interview child 4. inform the authorities 5. request forensic medical consultation (option only for physicians) 6. none of the above applies |
| Does your institution have specific instructions on how to respond to suspected child abuse and neglect? | 1. yes, there are specific instructions in my institution 2. no, there are no specific instructions in my institution 3. I am not aware of specific instructions in my institution |
| Do you think that a generally applicable guideline for reactions in such suspected cases could have a positive effect? | 1. yes 2. no 3. unsure |
| Did the duty of confidentiality influence you in your actions? | 1. yes 2. no 3. unsure |
| In which scenario does a breach of confidentiality occur? Multiple answers possible  Sharing information with … | 1. colleagues 2. parents 3. police 4. youth welfare office 5. other 6. none of the above |
| Do you think that the topic of child abuse and neglect is a taboo subject in your professional setting? | 1. yes 2. no 3. unsure |
| How do you think the topic of child protection should be discussed in public? | 1. intense 2. less intense 3. neutral 4. less 5. not at all |
| Have you attended any training on child abuse and neglect? Multiple answers possible | 1. yes, voluntarily 2. yes, during studies 3. yes, during working life 4. no |
| Should there be continuous education on this topic after studies? | 1. yes 2. no 3. unsure |
| Should the topic of child abuse and neglect be a mandatory part of the training of future pediatricians and teachers? | 1. yes 2. no 3. unsure |
| Do you feel adequately informed about child abuse and neglect? | 1. yes 2. no 3. unsure |
| Do you wish more information about child abuse and neglect? | 1. yes 2. no 3. unsure |
| By whom should this information be provided? | 1. psychologists 2. youth welfare office 3. physicians 4. school 5. health department 6. others |
